# Automatic Detection of Radiographic Alveolar Bone Loss in Bitewing and Periapical Intraoral Radiographs Using Deep Learning Technology: A Preliminary Evaluation

**DOI:** 10.3390/diagnostics15050576

**Published:** 2025-02-27

**Authors:** Amjad AlGhaihab, Antonio J. Moretti, Jonathan Reside, Lyudmila Tuzova, Yiing-Shiuan Huang, Donald A. Tyndall

**Affiliations:** 1 Department of Maxillofacial Surgery and Diagnostic Sciences, College of Dentistry, King Saud bin Abdulaziz University for Health Sciences, Riyadh 11481, Saudi Arabia; 2 Department of Diagnostic Sciences, Oral and Maxillofacial Radiology, Adams School of Dentistry, University of North Carolina at Chapel Hill, Chapel Hill, NC 27599, USA; yiingshiuanhuang@gmail.com (Y.-S.H.); don_tyndall@unc.edu (D.A.T.); 3 King Abdullah International Medical Research Center, Ministry of National Guard Health Affairs, Riyadh 11481, Saudi Arabia; 4 Department of Periodontology, Endodontics and Dental Hygiene Adams School of Dentistry, University of North Carolina at Chapel Hill, Chapel Hill, NC 27599, USA; antonio_moretti@unc.edu (A.J.M.); residedds@gmail.com (J.R.); 5 Denti.AI Technology Inc., Toronto, ON M5R 3K5, Canada; ltuzova@denti.ai

**Keywords:** alveolar bone loss, artificial intelligence, deep learning, dental digital radiography

## Abstract

**Background/Objective:** Periodontal disease is a prevalent inflammatory condition affecting the supporting structures of teeth, with radiographic bone loss (RBL) being a critical diagnostic marker. The accurate and consistent evaluation of RBL is essential for the staging and grading of periodontitis, as outlined by the 2017 AAP/EFP Classification. Advanced tools such as deep learning (DL) technology, including Denti.AI, an FDA-cleared software utilizing convolutional neural networks (CNNs), offer the potential for enhancing diagnostic accuracy. This study evaluated the diagnostic accuracy of Denti.AI for detecting RBL in intraoral radiographs. **Methods:** A dataset of 39 intraoral radiographs (22 periapical and 17 bitewing), covering 316 tooth surfaces (123 periapical and 193 bitewing), was selected from a de-identified pool of 500 radiographs provided by Denti.AI. RBL was assessed using the 2017 AAP/EFP Classification. A consensus panel of three board-certified dental specialists served as the reference standard. Performance metrics, including sensitivity, specificity, positive predictive value (PPV), negative predictive value (NPV), accuracy, and mean absolute error (MAE), were calculated. **Results:** For periapical radiographs, Denti.AI achieved a sensitivity of 76%, specificity of 86%, PPV of 83%, NPV of 80%, and accuracy of 81%, with an MAE of 0.046%. For bitewing radiographs, sensitivity was 65%, specificity was 90%, PPV was 88%, NPV was 70%, and accuracy was 76%, with an MAE of 0.499 mm. **Conclusions:** Denti.AI demonstrated clinically acceptable performance in detecting RBL and shows potential as an adjunctive diagnostic tool, supporting clinical decision-making. While performance was robust for periapical radiographs, further optimization may enhance its accuracy for bitewing radiographs.

## 1. Introduction

Periodontal Disease is an inflammatory pathological condition involving the periodontium that surrounds the teeth [1]. The Global Burden of Disease Study (GBD, 1990–2010) stated that severe periodontitis is the sixth most common disease worldwide [2]. Approximately 42% of dentate US adults aged 30 years or older had periodontitis, as depicted in a nationally representative study [3]. The diagnosis of periodontal disease requires a high level of clinical examination skills, and radiographs play an essential role in assessing alveolar bone levels, supplementing clinical examination [4].

The American Academy of Periodontology (AAP) evidence consensus statement indicates that a two-dimensional (2D) full-mouth radiographic series, along with clinical probing, is the gold standard for the radiographic evaluation of periodontal structures [5,6]. In 2017, the AAP and the European Federation of Periodontology (EFP) introduced a new multi-dimensional staging and grading system for periodontitis classification. It offers a thorough framework for clinicians to evaluate the severity, spread, and complexity of periodontitis, the progression rate of disease, and the anticipated response to treatment. Initial staging is determined using clinical attachment loss (CAL); however, if CAL is not available, radiographic bone loss (RBL) serves as a suitable alternative. Grading indicates disease progression based on direct or indirect evidence. When direct evidence is unavailable, progression is estimated by dividing RBL by the patient’s age [7,8]. Radiographic interpretation of RBL is therefore essential, as it plays a pivotal role in staging and grading periodontitis. With the increasing complexity of periodontal diagnosis and time constraints in clinical practice, advanced tools such as deep learning (DL) technology are being explored to support clinicians in achieving more accurate and consistent assessments.

DL technology is a subset of machine learning that utilizes multilayered neural networks to perform tasks such as classification and segmentation. Convolutional neural networks (CNNs), a type of deep neural network, are particularly well-suited for image analysis, as they can identify and learn features within images from input data, allowing the system to accurately classify and segment visual information [9]. The Denti.AI software (Denti.AI Technology Inc., Toronto, ON, Canada) (Version 1.3, http://denti.ai, accessed on 6 November 2023) is an FDA-cleared and commercially available software that utilizes CNNs to automate diagnostic tasks [10], including tooth numbering [11], caries detection, the identification of periapical lesions [12], and the assessment of radiographic bone levels. While Denti.AI software (Denti.AI Technology Inc., Toronto, ON, Canada) (Version 1.3, http://denti.ai, accessed on 6 November 2023) demonstrates capability in these areas, it has not been specifically tested for detecting RBL on intraoral radiographs.

Several studies have explored the potential of DL models for detecting RBL in periapical radiographs, with varying results across sensitivity, specificity, and overall accuracy [13,14,15,16,17,18,19,20,21,22,23,24,25,26,27]. Danks et al. (2021) used a CNN with hourglass architecture and reported an RBL error rate of 10.69% [17], while Lee et al. (2022) applied a U-Net CNN, achieving high performance with sensitivity ranging from 80% to 93% and specificity from 86% to 99% [18]. Chang et al. (2022) implemented an InceptionV3 model and reported an accuracy of 87%, with sensitivity and specificity values of 86% and 88%, respectively [20], whereas Chen et al. (2023) achieved the highest reported accuracy of 97% using an ensemble CNN model, though external validation was not performed [22]. Dai et al. (2024) evaluated AlexNet and VGG16, with both models yielding strong performance metrics, particularly in sensitivity and accuracy [25]. Collectively, these studies underscore the potential of DL models for RBL detection, yet none of these studies evaluated the commercially available Denti.AI software (Denti.AI Technology Inc., Toronto, ON, Canada) (Version 1.3, http://denti.ai, accessed on 6 November 2023). Our study extends this body of work by being the first to assess a commercially available tool, Denti.AI software (Denti.AI Technology Inc., Toronto, ON, Canada) (Version 1.3, http://denti.ai, accessed on 6 November 2023), for RBL detection in both periapical and bitewing intraoral radiographs, providing practical insights into its clinical applicability.

## 2. Materials and Methods

### 2.1. Selection and Characteristics of Intraoral Radiographs

This study was approved by the Institutional Review Board (IRB 21-3251) and assessed the diagnostic accuracy of a pre-trained and validated DL Denti.AI software (Denti.AI Technology Inc., Toronto, ON, Canada) (Version 1.3, http://denti.ai, accessed on 6 November 2023) for detecting RBL in intraoral radiographs. The software was initially trained using approximately 1000 intraoral radiographs, which included both anterior and posterior periapical radiographs as well as bitewing radiographs. Each radiograph was annotated by three general dentists to cover an estimated total of 8000 tooth surfaces. In this study, “tooth surface” refers to either the mesial or distal side of a tooth, with each tooth having two surfaces. Information on the specific distribution of radiographs in the training and validation sets and the experience levels of the general dentists who annotated them is limited, as these aspects were handled by an external group prior to the present study.

The primary investigator (AA) accessed a de-identified dataset of 500 randomly collected intraoral radiographs from Denti.AI company (Denti.AI Technology Inc., Toronto, ON, Canada), sourced from various North American insurance providers. From this pool, 100 radiographs were initially chosen, adhering to inclusion criteria for adults and acceptable diagnostic quality. Radiographs were excluded if they exhibited contact overlaps, poor angulation, supernumerary teeth, primary dentition, or the presence of implants. The final testing set comprised 39 radiographs: 22 periapical radiographs (18 posterior, 4 anterior), covering 74 teeth (123 surfaces), and 17 bitewing radiographs, covering 108 teeth (193 surfaces). The reporting of this study followed the Standards for Reporting Diagnostic Accuracy Studies (STARDs) 2015 guidelines [28].

### 2.2. Reference Standard, Annotation Protocol, and Radiographic Bone Loss Thresholds

Annotations of radiographic landmarks performed by a consensus panel served as the reference standard for the determination of RBL and against which the model prediction was tested. The consensus panel consisted of three American board-certified dental specialists; one oral and maxillofacial radiologist and two periodontists. Annotations were performed first by the periodontists, and any differences were reconciled by the oral and maxillofacial radiologist. The interobserver agreement was assessed using Cohen’s Kappa, yielding values of 0.69 for periapical radiographs and 0.83 for bitewing radiographs, indicating moderate and strong agreement, respectively [29].

The annotation process involved identifying key anatomical landmarks essential for calculating bone levels, as defined below:

1. C (CEJ); Cementoenamel junction, where enamel and cementum meet [30].
2. M (restoration margin)—the restoration boundary—used if the CEJ is obscured by a restoration [31].
3. Bone level (B); distance between the CEJ and the most apical location of the alveolar bone margin.
4. Root apex (R); in multi-rooted teeth, the leftmost and rightmost apices were annotated.

These landmarks were used to measure horizontal and vertical RBL, where RBL was defined in accordance with the 2017 AAP/EFP guidelines. For bitewing radiographs, RBL was defined as a distance of ≥2 mm between the CEJ and the most apical level of alveolar bone. For periapical radiographs, RBL was defined as present if the bone loss-to-root length ratio was ≥15% [7,8]. Table 1 details the RBL ranges across the sampled surfaces, categorized as per the 2017 AAP/EFP Classification of Periodontal Diseases guidelines. Due to the small sample size, RBL was recorded as either present or absent.

### 2.3. Workflow of the Deep Learning Software

The Denti.AI software (Denti.AI Technology Inc., Toronto, ON, Canada) (Version 1.3, http://denti.ai, accessed on 6 November 2023) workflow consists of multiple models, each utilizing CNN frameworks. The tooth detection model, based on the Faster-RCNN architecture, identifies and defines bounding boxes around each tooth in the radiograph. Following this, the tooth numbering model applies the ResNet CNN architecture to assign a unique number to each detected tooth. For bone level detection, the model applies Feature Pyramid Network (FPN) architecture combined with ResNet CNN to locate key anatomical landmarks (CEJ, restoration margin, bone level, and root apex) on the mesial and distal surfaces of each tooth. The FPN architecture includes two heads—one for determining key-point coordinates and another for evaluating their confidence levels. The software then calculates bone level measurements, including distances from the CEJ to the bone level and from the CEJ to the root apex, and computes the ratio of these distances.

### 2.4. Confusion Matrix Components and Statistical Analysis

The performance of the Denti.AI software (Denti.AI Technology Inc., Toronto, ON, Canada) (Version 1.3, http://denti.ai, accessed on 6 November 2023) was evaluated using sensitivity, specificity, accuracy, positive predictive value (PPV), negative predictive value (NPV), and mean absolute error (MAE). The definitions of the confusion matrix components and the metrics’ definitions and formulas are outlined in Table 2. MAE was reported in millimeters for bitewing radiographs and as a percentage for periapical radiographs, reflecting the average deviation of the model’s predictions from the reference standard. Given the exploratory nature of this study, the sample size was determined based on resource availability, and no power analysis was conducted. This study is intended to provide preliminary data for future, larger-scale research.

## 3. Results

Of the 123 periapical surfaces, 56 were identified as having RBL, while 98 of the 193 bitewing surfaces exhibited RBL according to the reference standard. Table 3 and Table 4 display the confusion matrices for detecting RBL in periapical and bitewing radiographs, respectively.

Table 5 summarizes the sensitivity, specificity, PPV, NPV, accuracy, and MAE for RBL detection in both periapical and bitewing radiographs.

**Figure 1:**
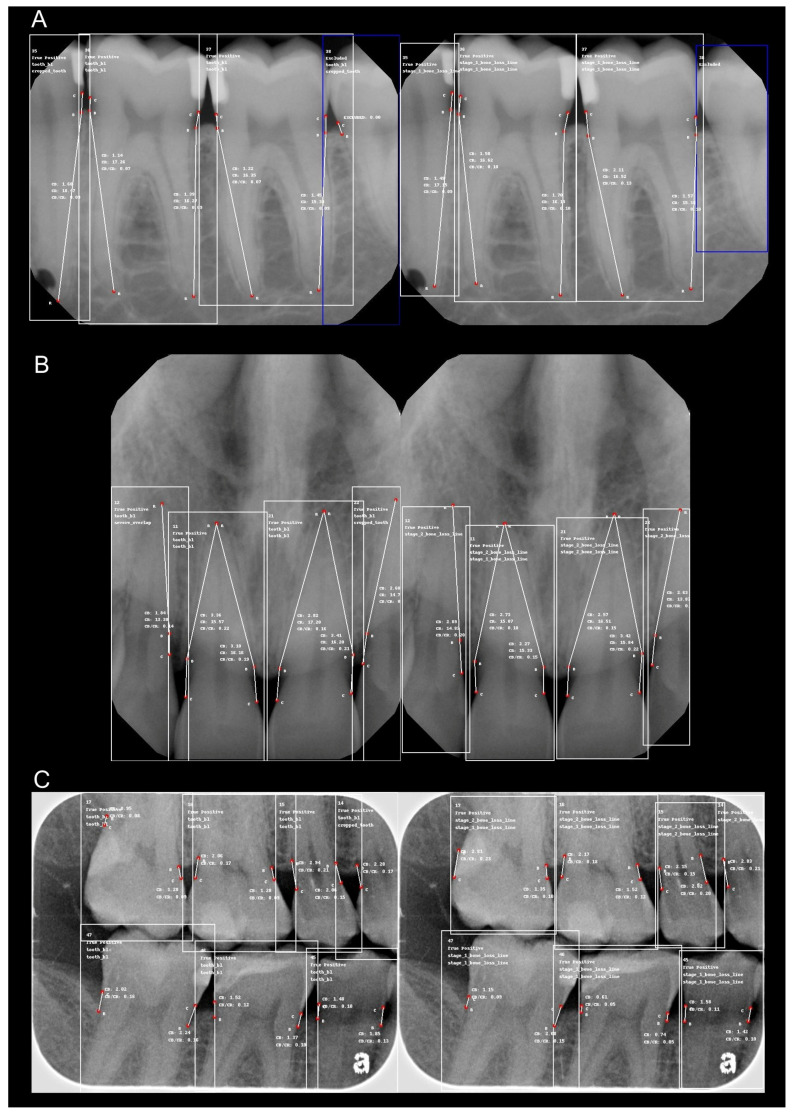
Comparative samples of radiographic bone loss (RBL) detection: reference standard versus model predictions. Images on the left represent the reference standard annotations, while images on the right display model-predicted points and measurements. (**A**) Posterior periapical radiographs, (**B**) anterior periapical radiographs, and (**C**) bitewing radiographs. Each image illustrates key anatomical landmarks, including the cementoenamel junction (CEJ), bone level, and root apex, used to assess RBL. In periapical radiographs, the CEJ is labeled as (C), bone level as (B), and root apex as (R), while in bitewing radiographs, only (C) and (B) are annotated.

Figure 1 presents comparative examples, illustrating the reference standard (left) and model predictions (right) for key points and measurements in posterior periapical radiographs (a), anterior periapical radiographs (b), and bitewing radiographs (c).

## 4. Discussion

This study aimed to evaluate the standalone efficacy of a deep learning-based Denti.AI software (Denti.AI Technology Inc., Toronto, ON, Canada) (Version 1.3, http://denti.ai, accessed on 6 November 2023) in RBL detection. For periapical radiographs, the model achieved a sensitivity of 76%, specificity of 86%, PPV of 83%, NPV of 80%, and an overall accuracy of 81%, with an MAE of 0.046%. In bitewing radiographs, the model demonstrated a sensitivity of 65%, specificity of 90%, PPV of 88%, NPV of 70%, and accuracy of 76%, with an MAE of 0.499 mm. PPV and NPV differ from sensitivity and specificity in that they depend on the disease prevalence, while the latter are independent of disease prevalence [32]. Given the high prevalence of periodontitis in the United States of America [3], reporting PPV may offer more practical insights than sensitivity, as it better reflects the predictive accuracy of the tool in real-world clinical settings.

The STARD 2015 guidelines provide standards for reporting diagnostic accuracy studies but do not define explicit cutoff points for what constitutes “good” or “acceptable” accuracy. Instead, they emphasize selecting and interpreting metrics based on the clinical context and the study goals. Diagnostic testing encompasses a variety of clinical roles, ranging from serving as a primary tool to acting as a triage or add-on aid. When test results are classified as positive or negative, a confusion matrix (2 × 2 table) estimates sensitivity, specificity, and predictive values by comparing the index test results to the reference standard. For tests with multiple possible outcome values, a threshold is required to define a positive result. A receiver operating characteristic (ROC) curve displays sensitivity and specificity across different thresholds, with the area under the curve (AUC) indicating overall diagnostic accuracy [28]. In our study, with binary outcomes (RBL present or absent), we used sensitivity, specificity, and predictive values and provided the confusion matrix (Table 3 and Table 4). Furthermore, we believe DL technology should be employed as an adjunctive instrument rather than as a substitute for clinical judgment in the detection of RBL. With an accuracy of 81% for periapical radiographs and 76% for bitewing radiographs, this Denti.AI software (Denti.AI Technology Inc., Toronto, ON, Canada) (Version 1.3, http://denti.ai, accessed on 6 November 2023) demonstrates clinically acceptable performance as an adjunct tool. Additionally, we evaluated the MAE to offer a nuanced understanding of the model’s performance. MAE, calculated as the average of the absolute errors between model predictions and reference measurements, reflects how closely the model’s predictions align with the reference standard, with lower values indicating greater accuracy [33]. The MAE of 0.046% for periapical radiographs and 0.499 mm for bitewing radiographs was considered clinically insignificant, suggesting that the model’s predictions closely align with the reference standard. As an adjunctive aid, DL could enhance clinical workflow by reducing the time needed for manual assessment through optimized radiographic analysis and automated identification of areas of concern. Furthermore, DL may help minimize variability among clinicians and promote consistency by offering a standardized screening approach. Lastly, DL has the potential to assist in training and guiding less experienced clinicians, enhancing their confidence and decision-making skills without replacing their essential role in diagnosis [34].

Table S1 summarizes the current literature on detecting RBL using DL technology in intraoral radiographs [35]. Numerous studies have evaluated the effectiveness of DL models in detecting RBL in periapical radiographs. Earlier studies used the previous periodontal classification system as the threshold for detecting RBL [13,21], while some provided insufficient details about their chosen thresholds [14,16], and more recent studies have adopted the 2017 AAP/EFP Classification of Periodontal Diseases as their RBL threshold [17,18,19,20,22,23,24,25,26,27]. Danks et al. (2021)’s hourglass architecture model showed an average RBL error of 10.69% compared to clinicians’ assessments [17]. In contrast, the present study demonstrated a significantly lower MAE of 0.046% for periapical radiographs despite using the same RBL thresholds. This difference may be due to variations in model architecture and reference standards, as Danks et al. (2021) used two periodontology residents [17], while our reference involved three board-certified specialists. Notably, Danks et al. (2021) did not report additional metrics recommended by the STARD 2015 guidelines for reporting diagnostic studies. Lee et al. (2022) tested a U-Net CNN for RBL detection showing high performance with sensitivity between 80% and 93%, specificity from 86% to 99%, and accuracy from 88% to 99% [18]. The higher performance likely stems from the use of a larger sample size and the integration of clinical periodontal diagnoses, which can improve accuracy. Chang et al. (2022) evaluated the InceptionV3 CNN model to detect RBL as a binary outcome [20] using a similar protocol, reference standard, and RBL thresholds as the present study. Chang et al. found an accuracy of 87%, sensitivity of 86%, specificity of 88%, PPV of 88%, and NPV of 86% [20]. The improved performance metrics may be due to the use of a larger sample size, which included 6219 tooth surfaces, although the exact size of the test set was not specified [20]. Additionally, Chen et al. (2024) evaluated the performance of two CNNs—U-Net and Mask-RCNN—in RBL detection in periapical radiographs [27]. The overall accuracy reported was 72.8% [27], which is lower than our model.

Chen et al. (2023) trained and validated an ensemble of CNN models, achieving an impressive accuracy of 97% [22], the highest reported to date. However, their model has not been externally tested. Ideally, the testing set should include radiographs from sources different from those used for training and validation to reduce potential bias. Dai et al. (2024) evaluated AlexNet and VGG16 for the detection of RBL in periapical radiographs based on the 2017 AAP/EFP Classification. AlexNet achieved 91.5% sensitivity and 87.2% accuracy, while VGG16 showed 84.7% sensitivity and 85.3% accuracy, both outperforming our model’s sensitivity of 76% and accuracy of 81%. These differences may reflect larger dataset sizes, architectural variations, and contextual clinical data. However, the present model demonstrated a specificity (86%) comparable to VGG16 (86.4%) and higher than AlexNet (77.9%) [25], underscoring its reliability in correctly identifying the absence of disease. Dujic et al. (2023) evaluated five open-source transformer networks on a test set of 3000 periapical radiographs, achieving accuracy rates between 83% and 85% without manual annotation of regions of interest, which they noted as a limitation [24]. Similarly, Hoss et al. (2023) assessed five CNNs using the same test set without manual annotation. Their accuracy ranged from 82.0% to 84.8%, with sensitivity between 88.8% and 90.7% and specificity from 66.2% to 71.2%, reporting the highest performance in mandibular anterior teeth [23]. In our study, we achieved a similar overall accuracy of 81% with a smaller, manually annotated sample. However, further analysis by jaw or tooth region (‘mandible vs. maxilla’ and ‘anterior vs. posterior’) was not feasible due to sample size limitations.

The interpretation of findings in the context of existing research requires careful consideration of methodological differences, as these can significantly influence results. The heterogeneity of methodologies across studies evaluating the performance of DL models for detecting RBL in periapical radiographs complicates direct comparisons, particularly when thresholds, case definitions, and reporting metrics vary. For example, Moran et al. (2020) reported RBL as a binary outcome without specifying the threshold [14]. Despite this, their ResNet CNN achieved a sensitivity of 75%, specificity of 73%, PPV of 73%, and NPV of 74% [14], while our ResNet CNN demonstrated slightly higher values, likely due to the added FPN architecture. FPN enhances object detection across scales by creating a feature pyramid, combining high-level, low-resolution features with low-level, high-resolution features via a top-down pathway and lateral connections. This enables the detection of objects of varying sizes by utilizing multi-scale feature representations [36]. Additionally, Chen et al. (2021) trained and validated a Faster R-CNN model to detect dental diseases, including periodontitis [15]. However, due to the lack of clinical data, “RBL” is a more accurate term than “periodontitis” which requires clinical assessment. Their validation showed PPV and sensitivity between 50% and 60% for RBL across severity levels (Mild: <1/3 root length; Moderate: 1/3–1/2 root length; Severe: >1/2 root length) [15]. Although our study achieved better results, direct comparison is difficult due to differences in definitions, metrics, and the absence of external testing. Lastly, Lee et al. (2018), Alotaibi et al. (2022), and Tsoromokos et al. (2022) evaluated different CNNs, focusing on posterior, anterior, and mandibular periapical radiographs, respectively [13,19,21]. Since our analysis did not separate anterior and posterior radiographs nor maxilla and mandible, direct comparison is not feasible. In summary, the variability in methodologies and metrics across studies complicates comparisons, requiring clinicians to interpret DL model performance cautiously.

Although a full-mouth radiographic series, including bitewing radiographs combined with clinical probing, is the gold standard for evaluating periodontal structures [5,6], only one study, to our knowledge, has assessed the performance of DL technology for detecting RBL in bitewing radiographs. Erturk et al. (2024) evaluated a YOLOv8 CNN on a test set of 350 bitewing radiographs, classifying RBL using the 2017 AAP/EFP Classification of Periodontal Diseases. Their model achieved an accuracy of 83%, PPV of 82%, sensitivity of 81%, and F1-score of 81% [26]. While their model outperformed ours in sensitivity and accuracy, our model achieved a higher PPV of 88% compared to their 82%. Notably, their approach classified radiographs rather than annotating landmarks and calculating RBL [26]. These differences in performance metrics likely reflect variations in model architecture, sample size, and training methodologies.

This study has several notable strengths. First, the model was tested on radiographs from diverse sources, specifically from various North American insurance companies, which helps to reduce potential model bias. Additionally, our reference standard was robust, established by three board-certified specialists—one oral and maxillofacial radiologist and two periodontists—ensuring a high level of expertise. Furthermore, we followed the 2017 AAP/EFP Classification of Periodontal Diseases for RBL thresholds and adhered to STARD 2015 guidelines for diagnostic accuracy reporting, which promotes transparency and facilitates comparability with other studies. Finally, by treating each tooth surface as an individual data point rather than averaging surfaces per radiograph, we enhanced precision in RBL detection and minimized potential sources of error.

Nevertheless, this study also has certain limitations. First, the small sample size served as preliminary data for assessing commercially available software, limiting generalizability. A larger dataset could enhance the model’s diagnostic accuracy by improving its ability to capture variability across different cases, reducing potential biases, and increasing the robustness of the findings. Additionally, variations in image quality, including differences in contrast, resolution, exposure settings, and angulation, could influence the model’s performance, particularly in cases of suboptimal image quality. This study focused solely on bone levels in 2D radiographs without incorporating clinical diagnoses or assessing other periodontal defects, such as furcation involvement. Moreover, the model was pre-trained and validated using datasets prepared by an external group of three general dentists with varying experience levels, while we conducted the testing phase using a reference standard set by three board-certified specialists. This difference in groups ideally calls for an assessment of interobserver agreement; however, this was not practically feasible. Clinically, this limitation may have a minimal impact, as our primary goal was to evaluate the model against what we consider the strongest reference standard. Such discrepancies in training, validation, and testing groups are a common challenge when testing clinically available software, which is often pretrained and validated by external teams prior to clinical use.

Future studies should address these limitations by using a larger sample size and incorporating clinical periodontal diagnoses for a more comprehensive assessment. Additionally, the inclusion of a broader range of periodontal defects, such as fenestration and dehiscence, could further expand the diagnostic capabilities of these models, as these findings remain underexplored [37]. Training models to assess both bone levels and periodontal defects may also offer insights into their capacity for simultaneous detection and classification. Furthermore, to enhance transparency and comparability, future studies should adhere to STARD 2015 guidelines. Finally, as new models continue to emerge, standardized testing protocols for assessing bone levels are essential, and comparative diagnostic studies examining the effectiveness of linear measurements from landmark detection versus segmentation could be a first step toward establishing a standardized protocol that aligns best with the reference standard.

## 5. Conclusions

Denti.AI software demonstrated clinically acceptable performance as an adjunctive tool for detecting RBL in intraoral periapical and bitewing radiographs, and further fine-tuning with a larger dataset could enhance its performance, particularly with bitewing radiographs.

## Figures and Tables

**Table 1:** Distribution of radiographic bone loss (RBL) across sampled surfaces according to the 2017 AAP/EFP Classification of Periodontal Diseases ^1^.

Threshold	<15% (Control Surfaces)	(Positive Surfaces)	
		15–33%	33–66%
Periapical Radiograph (*n* = 22 with 123 surfaces)	67	51	5
**Threshold**	**<2 mm (Control** **Surfaces)**	**(Positive Surfaces)**	
		**2–4 mm**	**≥5 mm**
Bitewing Radiograph (*n* = 17 with 193 surfaces)	95	88	10

^1^ For bitewing images, RBL was defined as a CEJ–bone distance of ≥2 mm, and for periapical images, as alveolar bone loss of ≥15% of root length [7,8]. For data analysis, surfaces were classified into two categories: “control surfaces” indicating the absence of RBL and “positive surfaces” indicating the presence of RBL.

**Table 2:** Confusion matrix components and statistical analysis metrics for deep learning model performance in detecting radiographic bone loss (RBL).

Category	Metric	Definition	Formula
Confusion Matrix Components	True Positive (TP)	Model correctly predicts >2 mm bone loss in bitewing radiographs and ≥15% bone loss in periapical radiographs.	N/A
	True Negative (TN)	Model correctly predicts <2 mm bone loss in bitewing radiographs and <15% bone loss in periapical radiographs.	N/A
	False Positive (FP)	Model predicts >2 mm bone loss in bitewing radiographs and ≥15% bone loss in periapical radiographs, but the reference standard does not.	N/A
	False Negative (FN)	Model predicts <2 mm bone loss in bitewing radiographs and <15% bone loss in periapical radiographs, but the reference standard does not, or the model does not make a prediction.	N/A
Statistical Analysis	Sensitivity	Probability that the model correctly detects bone loss when it is present.	Sensitivity = P (T+|D +) = TP/(TP + FN)
	Specificity	Probability that the model correctly identifies the absence of bone loss when it is not present.	Specificity = P (T-|D-) = TN/(TN + FP)
	Positive Predictive Value (PPV)	Probability that bone loss is present when the model predicts it.	PPV = P (D+|T+) = TP/(TP + FP)
	Negative Predictive Value (NPV)	Probability that bone loss is absent when the model predicts no bone loss.	NPV = P (D-|T-) = TN/(TN + FN)
	Accuracy	Overall correctness of the model’s predictions.	Accuracy = TP + TN/TP + TN + FP + FN
	Mean Absolute Error (MAE)	Average of the absolute errors between model predictions and reference measurements.	$MAE=\frac{\sum_{i=1}^{n}\left|y_{i}-x_{i}\right|}{n}$ MAE=mean absolute error $y_{i}=prediction$ $x_{i}=true\;value\;$ n=total number of data point

**Table 3:** Confusion matrix comparing Denti.AI model predictions against the reference standard for the detection of radiographic bone loss (RBL) in periapical radiographs (*n* = 22, 123 surfaces).

		Reference Standard (Consensus Panel)		Total Number of Surfaces
		RBL	No RBL	
Denti.AI Prediction	RBL	44 (35.8%)	9 (7.3%)	53 (43.1%)
	No RBL	14 (11.4%)	56 (45.5%)	70 (56.9%)
Total Number of Surfaces		58 (47.2%)	65 (52.8%)	123 (100%)

Note: Values represent frequencies, with percentages out of the total in parentheses.

**Table 4:** Confusion matrix comparing Denti.AI model predictions against the reference standard for the detection of radiographic bone loss (RBL) in bitewing radiographs (*n* = 17, 193 surfaces).

		Reference Standard (Consensus Panel)		Total Number of Surfaces
		RBL	No RBL	
Denti.AI Prediction	RBL	66 (34.2%)	9 (4.7%)	75 (38.9%)
	No RBL	36 (18.7%)	82 (42.5%)	118 (61.1%)
Total Number of Surfaces		102 (52.8%)	91 (47.2%)	193 (100%)

Note: Values represent frequencies, with percentages out of the total in parentheses.

**Table 5:** Performance metrics for the detection of radiographic bone loss (RBL) in periapical and bitewing radiographs.

Metrics	Bitewing Radiographs (*n* = 17, 193 Surfaces)	Periapical Radiographs (*n* = 22, 123 Surfaces)
Sensitivity	65% (95% CI: 58.3–71.7%)	76% (95% CI: 68.5–83.5%)
Specificity	90% (95% CI: 85.8–94.2%)	86% (95% CI: 79.9–92.1%)
PPV	88% (95% CI: 83.4–92.6%)	83% (95% CI: 76.4–89.6%)
NPV	70% (95% CI: 63.5–76.5%)	80% (95% CI: 73.0–87.1%)
Accuracy	76% (95% CI: 69.9–82.0%)	81% (95% CI: 74.1–87.9%)
MAE	0.499 mm	0.046%

Note: Sensitivity, specificity, PPV, NPV, and accuracy include 95% confidence intervals (CIs). MAE units are in millimeters (mm) for bitewing radiographs and in percentage (%) for periapical radiographs, as explained in the methodology.

## Data Availability

The data that support the findings of this study are available from the corresponding author upon reasonable request.

## Supplementary Materials

The following supporting information can be downloaded at: https://www.mdpi.com/article/10.3390/diagnostics15050576/s1, Table S1: Summary of Studies (N = 15) on Deep Learning (DL) for Detecting Radiographic Bone Loss (RBL) in Intraoral Radiographs.
