# Supplementary material for: Automatic Detection of Radiographic Alveolar Bone Loss in Bitewing and Periapical Intraoral Radiographs Using Deep Learning Technology: A Preliminary Evaluation
Source: Diagnostics (Basel). 2025 Feb 27;15(5):576. doi: 10.3390/diagnostics15050576 (PMC11899607; doi:10.3390/diagnostics15050576)
Supplement: Supplementary file 1 [file diagnostics-15-00576-s001.zip › diagnostics-3477464-supplementary.pdf]

Table S1. Summary of Studies (N=15) on Deep Learning (DL) for Detecting Radiographic Bone Loss (RBL) in Intraoral Radiographs.

Authors and Year	Country of Origin	Type of Intraoral Radiograph & Testing Sample Size	AI Architecture	Task	Reference Standard	RBL Threshold †	STARD 2015 Guidelines Reported	Performance Metrics ‡
**Lee et al., 2018 [13]**	South Korea	**Periapical** (posterior only); ***348 radiographs***	VGG-19 CNN	Detection and classification	Three board-certified periodontists	Classification Criteria: Healthy: CAL < 3 mm; Moderate: bleeding on probing, CAL < 6 mm, bone loss < 4 mm; Severe: CAL > 6 mm, bone loss > 4 mm	No	**Accuracy**: 81% (premolars), 76.7% (molars)
**Moran et al., 2020 [14]**	Brazil	**Periapical**; ***104 radiographs***	ResNet, Inception	Detection	Two experts (general dentist and oral radiologist)	Presence or absence; no specific numerical threshold	No	**ResNet**: **Sensitivity**: 75%, **Specificity**: 73.1%, **PPV**: 73.6%, **NPV**: 74.5%, **AUC-ROC**: 0.864; **Inception**: **Sensitivity**: 92.3%, **Specificity**: 71.1%, **PPV**: 76.2%, **NPV**: 90.2%, **AUC-ROC**: 0.860
**Chen et al., 2021 [15]**	China	**Periapical**; only trained and validated the model with a total of ***2900 radiographs***	Faster Region-Based CNN	Detection and classification of RBL in addition to two other dental diseases	One dentist	Mild: <1/3 root length; Moderate: 1/3–1/2 root length; Severe: >1/2 root length	No	**PPV** and **sensitivity** generally ranged between 50% and 60% for RBL across severity levels§¶
**Khan et al., 2021 [16]**	Saudi Arabi, Pakistan, UK	**Periapical**; ***30 radiographs***	Multiple CNNs (U-Net, Xnet, SegNet, U-Net + Densenet121)	Detection and segmentation of RBL in addition to two other dental diseases	Three specialists (two oral pathologists, including one board-certified, and one endodontist)	Not mentioned	No	**U-Net architecture** outperformed the other AI architectures with a **Mean IoU** of 0.402 and **Dice coefficient** of 0.453
**Danks et al., 2021 [17]**	UK	**Periapical**; test size unspecified (total 340 radiographs across all data sets)	Hourglass networks	Detection and classification	Two periodontology residents	2017 AAP/EFP Classification of Periodontal Diseases	No	**MAE**: 10.69% ± 9.15%; **Accuracy**: 58%
**Lee et al., 2022 [18]**	USA	**Periapical**; ***139 radiographs + 644*** "***additional dataset***"	U-Net, ResNet-34 CNNs	Detection and classification	Three examiners (two periodontists and one periodontal resident)	2017 AAP/EFP Classification of Periodontal Diseases	No	**Stage I RBL:** (**Sensitivity:** 82%, **Specificity:** 97%, **Accuracy:** 91%, **AUROC:** 89%); **Stage II RBL:** (**Sensitivity:** 93%, **Specificity:** 86%, **Accuracy:** 88%,

Table S1. Summary of Studies (N=15) on Deep Learning (DL) for Detecting Radiographic Bone Loss (RBL) in Intraoral Radiographs.

								**AUROC:** 90%); **Stage III RBL:** (**Sensitivity:** 80%, **Specificity:** 99%, **Accuracy:** 99%, **AUROC:** 90%); **Normal:** (**Sensitivity:** 96%, **Specificity:** 100%, **Accuracy:** 99%, **AUROC:** 98%)
**Tsoromokos et al., 2022 [19]**	The Netherlands	**Periapical** (mandible only); ***140 tooth surfaces***	CNN (unspecified)	Detection	One periodontist	2017 AAP/EFP Classification of Periodontal Diseases	No	**MAE**: 4.7%
**Chang et al., 2022 [20]**	USA	**Periapical**; test size unspecified (total 2362 radiographs with 6219 tooth surfaces)	InceptionV3 CNN	Detection and classification	Three board-certified periodontists	2017 AAP/EFP Classification of Periodontal Diseases	No	**Sensitivity**: 86%, **Specificity**: 88%, **PPV**: 88%, **NPV**: 86%, **Accuracy**: 87%
**Alotaibi et al., 2022 [21]**	Saudi Arabia	**Periapical** (anterior only); ***173 radiographs***	VGG-16	Detection and classification	Three examiners (one periodontist)	Classification by the International Workshop for Periodontal Diseases (1999)	No	**Multi-class: Mild:** (**Sensitivity:** 45%, **PPV:** 45%, **F1:** 45%), **Moderate:** (**Sensitivity:** 60%, **PPV:** 52%, **F1:** 56%), **Severe:** (**Sensitivity:** 45%, **PPV:** 83%, **F1:** 59%), **Normal:** (**Sensitivity:** 70%, **PPV:** 70%, **F1:** 70%), **Accuracy:** 59%; **Binary: Abnormal:** (**Sensitivity:** 77%, **PPV:** 73%, **F1:** 75%), **Normal:** (**Sensitivity:** 69%, **PPV:** 73%, **F1:** 71%), **Accuracy:** 73%.§¶
**Chen et al., 2023 [22]**	Taiwan	**Periapical**; only trained and validated the model with a total of 8000 radiographs	YOLOv5, VGG-16, U-Net	Detection and segmentation	Five dentists with periodontology and radiology training	2017 AAP/EFP Classification of Periodontal Diseases	STROBE	**Accuracy**: 97%
**Hoss et al., 2023 [23]**	Germany	**Periapical**; ***3000 radiographs***	Five CNNs (ResNet-18, MobileNet V2, and three configurations of ConvNeXT (small, base, and large)	Classification	Categorized by four graduate dentists, then reviewed by three experienced dentists; no annotation performed	2017 AAP/EFP Classification of Periodontal Diseases	Yes	**Overall:** (**Sensitivity:** 88.8%–90.7%, **Specificity:** 66.2%–71.2%, **Accuracy:** 82.0%–84.8%, **AUC:** 0.884–0.913)
**Dujic et al., 2023 [24]**	Germany	**Periapical**; ***3000 radiographs***	Five open-source transformer networks (ViT-	Classification	Categorized by four graduate dentists, then reviewed by three experienced	2017 AAP/EFP Classification of Periodontal Diseases	Yes	**Overall:** (**Accuracy:** 83.4–85.2%, **AUC:** 0.899–0.918); **Lower Anterior Teeth:** (**Accuracy:** 94.1–96.7%, **AUC:** 0.944–0.970);

Table S1. Summary of Studies (N=15) on Deep Learning (DL) for Detecting Radiographic Bone Loss (RBL) in Intraoral Radiographs.

			base, ViT-large, BEiT-base, BEiT-large, and DeiT-base)		dentists; no annotation performed			**Upper Anterior Teeth:** (**Accuracy:** 86.7–90.2%, **AUC:** 0.948–0.958); **Lower Posterior Teeth:** (**Accuracy:** 85.6–87.2%, **AUC:** 0.913–0.937); **Upper Posterior Teeth:** (**Accuracy:** 78.1–81.0%, **AUC:** 0.851–0.875)
**Dai et al., 2024 [25]**	China	**Periapical**; ***2780 radiographs***	AlexNet, VGG16, ResNet18 CNNs	Detection and classification	Three periodontists	2017 AAP/EFP Classification of Periodontal Diseases	No	**AlexNet:** (**Sensitivity:** 91.5%, **Specificity:** 77.9%, **Accuracy:** 87.2%); **VGG16:** (**Sensitivity:** 84.7%, **Specificity:** 86.4%, **Accuracy:** 85.3%)
**Erturk et al., 2024 [26]**	Turkey	**Bitewing**; ***350 radiographs***	YOLOv8	Detection and classification	One dentist (oral radiology experience)	2017 AAP/EFP Classification of Periodontal Diseases	No	**Sensitivity:** 81%, **PPV:** 82%, **Accuracy:** 83%, **F1-score:** 81%
**Chen et al., 2024 [27]**	Taiwan	**Periapical**; ***336 radiographs***	U-Net, Mask-RCNN	Detection segmentation, and classification	Three board-certified periodontists	2017 AAP/EFP Classification of Periodontal Diseases	No	**Total Diagnostic Accuracy:** 72.8%; **Diagnostic Accuracy Per Stage: Stage I:** 64.2%, **Stage II:** 74.3%, **Stage III:** 94.0%; **Pearson Correlation Coefficient:** 0.828

Abbreviations:

- **AAP/EFP**: American Academy of Periodontology/European Federation of Periodontology
- **AI**: Artificial Intelligence
- **AUC**: Area Under the Curve
- **AUC-ROC**: Area Under the Receiver Operating Characteristic Curve
- **CAL**: Clinical Attachment Loss
- **CNN**: Convolutional Neural Network
- **IoU**: Intersection over Union
- **MAE**: Mean Absolute Error
- **NPV**: Negative Predictive Value
- **PPV**: Positive Predictive Value
- **RBL**: Radiographic Bone Loss
- **ROC**: Receiver Operating Characteristic
- **STARD**: Standards for Reporting Diagnostic Accuracy
- **STROBE**: Strengthening the Reporting of Observational Studies in Epidemiology
- **UK**: United Kingdom
- **USA**: United States of America

† Terms such as "periodontal bone loss" and "periodontally compromised teeth" have been standardized to "radiographic bone loss (RBL)" for consistency.

‡ Only results with objectives similar to our study are included from each referenced study.

§ "precision" has been changed to "positive predictive value (PPV)" and ¶ "recall" to "sensitivity" for consistency, as described by Sokolova & Lapalme (2009) and Powers (2011) [35].
